# P27 A case of unilateral scleritis and biopsy proven retinal vasculitis in an elderly woman with rheumatoid arthritis and previous retinal detachment surgery with silicone oil band

**DOI:** 10.1093/rap/rkad070.048

**Published:** 2023-09-27

**Authors:** Terry Kwong, Stephanie Young, John Downie, Svetlana Cherepanoff

**Affiliations:** St Vincents Clinic, Sydney, Australia; St George Private Hospital, Sydney, Australia; Concord Hospital, Sydney, Australia; Sydney Eye Hospital, Sydney, Australia; St Vincent's Hospital, Sydney, Australia

## Abstract

**Introduction:**

Scleritis is a serious and well recognised extra-articular manifestation of rheumatoid arthritis. Retinal vasculitis is a rare complication of rheumatoid arthritis. We reported a case of an 85-year-old woman with scleritis and biopsy-proven retinal necrosis and vasculitis. She could not tolerate traditional disease-modifying agents. Her scleritis and vasculitis did not respond to anti TNF and JAK inhibitors but settled with rituximab.

**Case description:**

Our patient is an 85-year-old woman who developed Rh F positive (Rh F 384, Normal < 14) rheumatoid arthritis at aged 70. She was treated with hydroxychloroquine and sulfasalazine but could not tolerate both. She required prednisone up to 10mg daily. She was treated with methotrexate with good control of her arthritis but she stopped taking methotrexate due to chest pain and bitter taste. Her arthritis was under control with leflunomide but she developed peripheral neuropathy. She was treated with prednisone 8mg daily between the age of 78 to 82 and her arthritis was stable. She has osteopenia and she was treated with denosumab.

She developed a flare of arthritis at the age of 82. She was treated with tofacitinib followed by adalimumab and subsequently with golimumab for 6 months. Her arthritis improved but she developed scleritis in her right eye while on golimumab. She had retinal detachment in her right eye at the age of 63 treated with vitrectomy and silicone oil with postoperative vision stable at hand motion.

Examination showed a patch of scleritis in her temporal and inferior sclera, with a scleral nodule superotemporally. There was initially no intraocular inflammation or new retinal changes. Her left eye did not show any scleritis or intraocular inflammation. She was treated with 2 subconjunctival injections of triamcinolone and topical steroids. Her scleritis improved but did not resolve.

Her scleritis worsened about 3 months after the presentation affecting the superonasal sclera. There was mild anterior chamber inflammation and she was found to have numerous pale subretinal nodules throughout her right fundus with some subretinal fluid. Her golimumab was stopped but she required prednisone up to 20mg a day to control her arthritis, right eye pain and scleritis.

**Discussion:**

She underwent a vitrectomy of her right eye to obtain a vitreous and retinal sample. A 2.5mm piece of retina was excised along with an underlying pale subretinal nodule. Ultimately, vision deteriorated to no perception of light, but the eye was comfortable, with the development of extensive subretinal fibrosis.

The retinal and vitreous biopsy consisted of 20ml of clear colourless fluid with occasional tissue fragments and a cell block was prepared. There was no evidence of lymphoma by light microscopy, immunohistochemistry or flow cytometry. Cultures were negative for bacterial or fungal infection. PCRs were negative for herpes or CMV infection. Histology of the retinal biopsy showed a focus of retinal necrosis in addition to retinal vasculitis, with intramural lymphocytes and neutrophils. On cytology, vitreous cellularity was high, consisting of macrophages, neutrophils, small and mildly enlarged lymphocytes, hyalocytes, microglia and occasional plasma cells. She had persistent pain in her right eye requiring moderately high dose of steroids and narcotics to control her pain. Her scleritis developed while she was on anti-TNF antibodies. Her retinal biopsy and subsequent course excluded viral retinitis or lymphoma.

She presented with a therapeutic dilemma as she has no vision in her right eye. However, scleritis is not only painful but is an indicator of underlying vasculitis. Scleritis is sight threatening and is associated with a very high cardiovascular mortality. Aggressive treatment is indicated to treat her scleritis and to control her pain and, more importantly, to prevent any involvement of her left eye and to prevent cardiovascular complications.

She was treated with IV rituximab after she had her 4th dose of COVID vaccine and had tixagevimab/cilgavimab cover. Her scleritis settled with IV rituximab. We were able to taper her prednisone to her previous stable dose of 8mg a day.

**Key learning points:**

This is a rare case of anterior scleritis with retinal necrosis in a patient with Rh F positive rheumatoid arthritis. She developed scleritis 14 years after the onset of her arthritis and while she was on golimumab. She had unilateral scleritis and retinal vasculitis in her right eye with previous surgery for detachment and silicone oil band inserted 15 years before the onset of her retinal vasculitis. Serial fundal examination and OCT showed the retinal lesions developed over a 3 months period. The histology of the retinal biopsy showed acute vasculitis with neutrophils and lymphocytes infiltration of vessel wall without granuloma. The role of silicone in the pathogenesis of scleritis needs to be explored and discussed in this meeting.

This case highlights the difficulty in treating elderly patients with severe rheumatoid arthritis with multiple drugs intolerances and multiple co-morbidities, especially during the COVID pandemic with use of rituximab.

Scleritis is a rare extra-articular manifestation of rheumatoid arthritis. A recent review showed the incidence of scleritis is only slightly reduced in the biologic era. However, the manifestation and complications of scleritis remain severe (80% with scleral necrosis) and similar in pre-biologic and post biologic era. Based on a recent review of 5 retrospective studies (1 post-biologic and 4 pre-biologic era), mortality remains unchanged in the post-biologic era.

Rituximab is an effective treatment in this patient with rheumatoid arthritis, scleritis and retinal vasculitis.

